# Current developments of the estimand concept

**DOI:** 10.1002/pst.2395

**Published:** 2024-04-27

**Authors:** Alexander Fierenz, Antonia Zapf

**Affiliations:** ^1^ Institute of Medical Biometry and Epidemiology University Medical Center Hamburg‐Eppendorf Hamburg Germany

**Keywords:** estimand, ICH E9 addendum, intercurrent event

## Abstract

Since the introduction of the estimand in therapeutical studies, several adaptions have been developed. This short article highlights the important aspects of the estimand concept. A literature research was conducted to identify different extensions to this framework. Different modified strategies for intercurrent events are presented, as well as examples of methods to implement the estimand in clinical studies. The article reflects that the estimand is an ongoing research field with further exploration.

In clinical trials, different methods can be used to handle protocol deviations. The estimation of the treatment effect should be aligned with the clinical question, which should guide the strategy how protocol deviations or predefined incidences should be taken into account. The estimand should be stated at the beginning to solve the problem of estimating the spurious treatment effect that is not consistent with the clinical question. Leuchs et al.^1^ made the first suggestion to use an estimand in clinical trials. They discriminated between treatment efficacy and effectiveness. Efficacy represents the treatment effect under optimal conditions, assuming full patient adherence. Effectiveness, on the other hand, considers real‐world scenarios when complete adherence may not be possible, for example, due to safety reasons. Another recommendation to report the treatment effect is the tripartite framework. It was proposed by Akacha et al.^2^ to answer the clinical questions from patients, regulators, and industry in the form of three combined scientific questions. These provide information on the percentage of non‐adherence to treatment due to safety (1) or lack of efficiency (2), together with the treatment effect for the adherers when they are able to take the treatment for the entire duration (3).

The International Council for Harmonisation (ICH) introduced the concept of estimands for therapeutic studies in 2017 for public consultation and adopted it in 2019.^3^ Since then, this has been a present topic of interest in the research of clinical studies. The ICH E9(R1) addendum defines an estimand in therapeutic studies as follows: “An estimand is a precise description of the treatment effect reflecting the clinical question posed by a given clinical trial objective. It summarises at a population level what the outcomes would be in the same patients under different treatment conditions being compared.”^3^ The estimand framework consists of five attributes used to describe and define the estimand, ensuring that it accurately reflects the clinical question.

Even before the introduction of the estimand, procedures existed to define the clinical question of interest before conducting a study. The PICO (Population, Intervention, Comparison, and Outcome) Framework provides a way to formulate these issues.^4^ The main difference to PICO, and therefore the main innovation of the estimands, is the identification and the selection of appropriate strategies for handling the intercurrent events (ICE) in the trial. These ICEs refer to incidents or protocol deviations that occur after treatment initiation and therefore, after randomisation. These events either influence the measurement result or make it impossible to determine it. Based on the chosen strategy, an ICE can lead to a non‐existing value which is not of interest to the trial objective. For this reason, the framework distinguishes between ICE and missing values. The estimand highlights the importance of these ICEs to ensure that the treatment conditions are comparable on the level of outcome measurement.^5^

In the first step of a clinical trial, the trial objective is translated to an estimand to specify what is to be estimated. Based on the estimand, a suitable method is derived as an estimator to calculate the numerical result (the estimate).^3^ The framework extends across the entire trial process, and enhances the interaction of the involved fields in formulating the objective, design, conduct, analysis, and interpretation of the trial.^3^

## 1 THE ESTIMAND CONCEPT

The estimand in therapeutic studies consists of five attributes. These are described by the ICH E9(R1)^3^ as follows:

- The **treatment** condition of interest and the alternative treatment. These can be both individual or a combination of interventions.
- The **population** of patients. This should represent the target population of interest for which the treatment effect is analysed.
- The **variable** (endpoint) to be determined for each patient. It defines the specific measurement which is of interest to analyse the effect of the treatment.
- The **population‐level summary** for the variable. This allows a comparison between the treatments.
- The **strategy for handling the intercurrent event**. This specifies which strategy is selected for the respective ICE. The ICH E9(R1) outlines five different strategies.

All these attributes define the estimand and allow a precise formulation of the clinical question of interest. As mentioned earlier, the first four attributes were also covered before introducing this framework. The innovation is the focus on the different ICEs. They have to be identified before the start of the study. Moreover, adequate handling must be considered in advance. The ICH E9(R1) addendum presents the following five strategies to deal with ICEs:

- *Treatment policy strategy*: The occurrence of the ICE is considered irrelevant to the interpretation of the treatment effect. Therefore, the variable of interest is used regardless of the occurrence of the ICE.
- *Hypothetical strategy*: A fictitious scenario is set up, in which the ICE would not have occurred. The variable of interest represents a hypothetical value that would have been measured if the ICE had no impact on the variable.
- *Composite variable strategy*: The ICE gives information about the outcome measurement of the patient. Therefore, their occurrence is combined with the variable of interest and adapts the definition of the outcome.
- *While on treatment strategy*: Measurements before and at the time of the ICE are important. Only the treatment effect before the occurrence of the ICE is of interest.
- *Principle stratum strategy*: The population of interest is selected based on whether an ICE would or would not occur on a particular treatment arm. The clinical question of interest for the treatment effect is exclusively stated in this principle stratum.

These strategies are only suggestions for handling the ICEs. It is also feasible to use supplementary strategies. The following section presents one possibility.

## 2 EXTENSION OF THE FRAMEWORK

Since the introduction of the estimand framework expansions, adaptions and applications have been developed. A literature research was conducted to find possible new attributes of the estimand and strategies to handle intercurrent events. The databases of PubMed and Web of Science were searched. A backward search was additionally performed. Articles with the term “estimand/s” in the title or keyword (Web of Science) and in combination with the terms “ICH” or “intercurrent event” in all fields were selected for the screening of the abstracts. The search included all results published until 31 May 2023. This resulted in a total of 309 results. After screening the abstracts, 36 papers were selected for full‐text review, which led to 28 selected papers dealing with further developments on estimands and their potential estimates. Reasons for exclusions were not covering a clinical topic, the application of an estimand in a specific clinical trial, comments to papers, or general information and other statements regarding the estimand framework. The following part will give a short overview of the results. Table S1 in the supplement presents the classified literature.

One type of estimand is the attributable estimand.^6^ This estimand is based on a determined mix of strategies. It differentiates between adversely related and unrelated ICEs to the randomised treatment. The adversely related events are deemed attributable, and thus, they are handled with a composite variable strategy. Events that do not relate adversely to the treatment are handled through a hypothetical strategy.

Other important aspects of estimands are the different strategies for the ICEs. Han and Zhou^7^ provide mathematical forms for the estimand. In their setting, they work out the differences between the strategies in the mathematical notation to help statisticians with estimation, and clinicians, sponsors, and regulators with interpretation.

The work of Qu and Lipkovich^8^ discusses different aspects of strategies for ICEs. One consideration concerns the treatment policy strategy. It is essential to distinguish between a treatment policy strategy and a dynamic treatment regime. A dynamic treatment regime defines a set of rules that apply to all patients and determines how the treatment plan is modified over time or in response to specific events. On the other hand, the treatment policy strategy is driven by patient‐specific treatment changes. Furthermore, Qu and Lipkovich suggest breaking down the hypothetical strategy into different sub‐strategies. The first one is the *controlled direct effect strategy*, which focuses on assessing the effect of interest in patients if they have adhered to their originally assigned treatment regime, regardless of the occurrence of any ICEs. The second is the *no treatment hypothetical strategy*, which assumes that patients who experience an ICE would not benefit from any treatment and therefore remain untreated since randomisation. The last strategy is the *partial treatment hypothetical strategy*, which considers that the subject was under treatment up to the occurrence of an ICE, and afterwards, the medication was discontinued.^8^

An additional strategy was introduced by Michiels^9^ to address a different clinical question. This strategy is known as the balanced strategy and relies on the assumption that ICEs are independent of the treatment arm. The balanced strategy focuses on the occurrence of ICEs in patients allocated to a specific randomisation group. The consideration of a group enables us to determine the frequency of occurrence of ICE for that arm. According to this strategy, if an ICE occurs in a patient in this group, it is assumed that a similar event would also happen if the same patient or a patient with similar characteristics were randomised to the comparator arm. For example, if a patient in the control group needs rescue medication, the balanced strategy assumes that the patient would also require rescue medication if randomised to the experimental group. This estimand defines the intention‐to‐treat effect, that is, the treatment effect resulting from treatment assignment, which is obtained when a patient on the active arm experienced an ICE only in case when the same ICE would also occur if the patient had been randomised to the control arm.^9^ In other words, the estimand adjusts the effect of treatment allocation by the effect of the ICE by considering a counterfactual scenario.

## 3 FURTHER DEVELOPMENTS

Providing strategies for handling the ICEs supports defining the trial objective. However, they do not allow the analysis of observed data. The methods for the main analysis, as well as their sensitivity analysis, should be aligned with the trial objective and accordingly with the stated estimand. Therefore, it is crucial to implement estimates for the various strategies. Ratich et al.^10^ compared different multiple imputation methods for the hypothetical strategy. The treatment policy estimand can be estimated with an imputation method based on off‐treatment data if sufficient data has been collected after the ICE.^11^ Further statistical methods are feasible to impute missing data occurring after the ICE within the framework of the treatment policy strategy.^12^ For the composite strategy, different methods were analysed, including single imputation and trimmed mean approach.^6^, ^13^, ^14^ Diverse estimators for the while alive strategy have been investigated, containing methods‐of‐moments and negative binomial methods,^15^ as well as nonparametric methods for recurrent events.^16^ To estimate the estimand for a principle stratum strategy, Magnusson et al.^17^ developed a Bayesian method. A summary of different principal stratum methods was further compiled.^18^

Beyond this, different applications of estimands were established in the literature. A table template was introduced for the Statistical Analyses Plan (SAP),^19^ or ideas were discussed on how to implement the estimand framework into a template for study protocols.^20^ Another paper considered various sample size approaches for studies with ICEs.^21^ Moreover, estimands for different study design settings were defined. They were specified for innovative complex designs like basket or platform trials,^22^ factorial trials,^23^ cluster‐randomised trials,^24^, ^25^ and adaptive designs.^26^ Estimands are also embedded in various settings, including time‐to‐event data,^27^ multiple‐dose and bioequivalence trials,^28^ observational studies,^29^ benefit–risk assessments,^30^ safety evaluations,^31^ and vaccine trials.^32^

To assess the extent to which the estimand is reported in clinical trials, or at least can be inferred from the available information, two reviews were conducted that found that the estimand is not yet adequately considered.^33^, ^34^ The estimand framework has meanwhile been introduced to a few guidelines. Among the guidelines issued by the European Medicine Agency (EMA), the guideline for clinical investigations of medicinal products of gout, the guideline for evaluation of medicinal products for bacterial infections, and the paediatric addendum on the guideline on clinical investigation of medical products for venous thromboembolic disease refer to the ICH E9(R1).^35^, ^36^, ^37^ The EMA guidelines trials involving registry‐based studies, evaluation of vaccines, treatment of Alzheimer's disease, treatment of Crohn's disease, and treatment of Ulcerative Colitis require that an estimand must be specified for clinical studies.^38^, ^39^, ^40^, ^41^, ^42^ According to points to consider letters, the estimand framework must be adopted in cases of major disruptions, such as the Covid pandemic or the war in the Ukraine.^43^, ^44^ Similar considerations are mentioned in some US Food and Drug Administration (FDA) guidelines, where they recommend predefining the estimand for developing drugs related to nonallergic rhinitis, allergic rhinitis, eosinophilic esophagitis, and smoking cessation.^45^, ^46^, ^47^, ^48^ The guideline for acute myeloid leukaemia also discusses particular aspects of the estimand framework.^49^ The guidelines addressing multiple endpoints, and adjustment for covariates in randomised clinical trials also touch upon the estimand.^50^, ^51^ Finally, the guideline ICH E8 prescribes that the estimand should be defined in the protocol and the treatment effect should account for the occurrence of ICE.^52^

To sum up, the estimand is a dynamic research field with several extensions beyond its original concept. Further work is needed to investigate and adapt this concept across various domains within therapeutic studies.

## FUNDING INFORMATION

This work was supported by the Deutsche Forschungsgemeinschaft (DFG) grant number ZA 687/6‐1.

## CONFLICT OF INTEREST STATEMENT

The authors declare no conflicts of interest.

## Supporting information


**Table S1.** Overview of the literature research.

## Data Availability

Data sharing is not applicable to this article as no new data were created or analysed in this study.
