# Supplementary material for: Current developments of the estimand concept
Source: Pharm Stat. 2024 Apr 27;23(6):864–9. doi: 10.1002/pst.2395 (PMC11602887; doi:10.1002/pst.2395)
Supplement: Supplementary file 1 — Table S1. Overview of the literature research. [file PST-23-864-s001.pdf]

authors	year	title	content
		**Strategies for handling intercurrent events**	
Darken et al.	2020	The attributable estimand: A new approach to account for intercurrent events	Attributable Estimand: chose the strategy based on the relation of the ICE to the outcome
Michiels et al.	2021	A novel estimand to adjust for rescue treatment in randomized clinical trials	Balanced strategy for ICE; ICE occurrence independent of treatment arm
Qu, Lipkovich	2021	Implementation of ICH E9 (R1): A Few Points Learned During the COVID-19 Pandemic	Analyses of the different strategies, and considerations on two ICEs at the same time
		**Methods for different strategies**	
Ratitch et al.	2018	Points to consider for analyzing efficacy outcomes in long-term extension clinical trials	Examples for estimates for the hypothetical strategy (multiple imputation)
Wang et al.	2018	An evaluation of the trimmed mean approach in clinical trials with dropout	Example for an estimate for the composite variable strategy (trimmed mean)
Keene	2019	Strategies for composite estimands in confirmatory clinical trials: Examples from trials in nasal polyps and steroid reduction	Examples for estimates for the composite variable strategy (imputation)
Magnusson et al.	2019	Bayesian inference for a principal stratum estimand to assess the treatment effect in a subgroup characterized by postrandomization event occurrence	Bayesian example for estimates for the principle stratum strategy
Roger et al.	2019	Treatment policy estimands for recurrent event data using data collected after cessation of randomised treatment	Estimand for treatment policy strategy
Wie et al.	2021	Properties of Two While-Alive Estimands for Recurrent Events and Their Potential Estimators	Examples for estimates for the while on treatment strategy
Lipkovich et al.	2022	Using principal stratification in analysis of clinical trials	Summary of different principal stratum methods
Mao	2022	Nonparametric inference of general while-alive estimands for recurrent events	Estimate for while-alive-strategy in recurrent event
Han, Zhou	2023	Defining estimands in clinical trials: A unified procedure	Mathematical Estimands for different ICE strategies
Wang et al. (b)	2023	Statistical methods for handling missing data to align with treatment policy strategy	Methods for missing data after ICE in treatment policy strategy
		**Application of estimands in different trial designs**	
Okwuokwnye, Peace	2019	Adaptive Design and the Estimand Framework	Estimands in therapeutic studies with adaptive designs
Rufibach	2019	Treatment effect quantification for time-to-event endpoints–Estimands, analysis strategies, and beyond	Estimands for time-to-event
Kilpatrick et al.	2020	Estimands and inference in cluster-randomized vaccine trials	Estimands for cluster-randomized therapeutic trials
Ring et al.	2020	The potential of the estimands framework for clinical pharmacology trials: Some discussion points	Estimands for multiple dose and bioequivalance trials
Collignon et al.	2022	Estimands and Complex Innovative Designs	Estimands for different designs (basket trial, platform trial,...)
Kahan et al.	2022	Estimands for factorial trials	Estimands in factorial trials (different methods in one trial)
Li et al.	2022	Estimands in observational studies: Some considerations beyond ICH E9 (R1)	Estrimands in observational studies / weighting of population
Fu et al.	2023	Application of estimand framework in ICH E9 (R1) to vaccine trials	Estimands for efficacy, safety and immunogenicity
Kahan et al.	2023	Estimands in cluster-randomized trials: choosing analyses that answer the right question	Estimands for cluster randomized trials
Ren et al.	2023	Estimand in benefit-risk assessment	Estimands for benefit and risks; Estimand for more than one measurement
Wang et al. (a)	2023	Application of estimand framework in ICH E9 (R1) to safety evaluation	Safety Estimands
		**Further estimand related topics**	
Fang et al.	2021	Sample Size Calculation When Planning Clinical Trials with Intercurrent Events	Consideration for sample sizes in the estimand concept
Kahan et al.	2021	Estimands in published protocols of randomised trials: urgent improvement needed	Review on Estimands reported or inferrable in trial protocols
Cro et al.	2022	Evaluating how clear the questions being investigated in randomised trials are: systematic review of estimands	Review on Estimands reported or determinable in RCTs
Kang et al.	2022	Incorporating estimands into clinical trial statistical analysis plans	Template for estimands which can be included in the statistical analysis plan
Lynggaard et al.	2022	Principles and recommendations for incorporating estimands into clinical study protocol templates	Recommendations how the estimand could be implemented in clinical study protocolls

Table S1 Overview of the literature research
